# Supplementary material for: Loss of CFTR Reverses Senescence Hallmarks in SARS-CoV-2 Infected Bronchial Epithelial Cells
Source: Int J Mol Sci. 2024 Jun 4;25(11):6185. doi: 10.3390/ijms25116185 (PMC11172982; doi:10.3390/ijms25116185)
Supplement: Supplementary file 1 [file ijms-25-06185-s001.zip › ijms-2946118-supplementary.pdf]

Supplementary materials

Supplementary Table S1. Percentage of p21-immunoreactive (IR) cells by light microscopy in mock- and SARS-CoV-2-infected WT and CFTR KO 16HBE14o- cells.

		Mock-infected		SARS-CoV-2 infected	
**hours**	**16HBE14o-**	**cells (*n*)**	**p21-IR cells, %**	**cells (*n*)**	**p21-IR cells, %**
3	WT	128	23	140	51
	CFTR KO	209	35	313	49
24	WT	101	32	135	60
	CFTR KO	219	51	244	37
48	WT	108	32	123	57
	CFTR KO	257	44	208	33
72	WT	133	28	134	48
	CFTR KO	266	44	318	39

Supplementary Table S2. Percentage of Ki67-immunoreactive (IR) cells by light microscopy in mock- and SARS-CoV-2-infected WT and CFTR KO 16HBE14o- cells.

		Mock-infected		SARS-CoV-2 infected	
**hours**	**16HBE14o-**	**cells (*n*)**	**Ki67-IR cells, %**	**cells (*n*)**	**Ki67-IR cells, %**
3	WT	141	87	115	72
	CFTR KO	295	91	358	86
24	WT	108	85	101	79
	CFTR KO	165	79	169	83
48	WT	138	89	111	86
	CFTR KO	221	75	191	87
72	WT	170	93	143	92
	CFTR KO	242	59	212	88

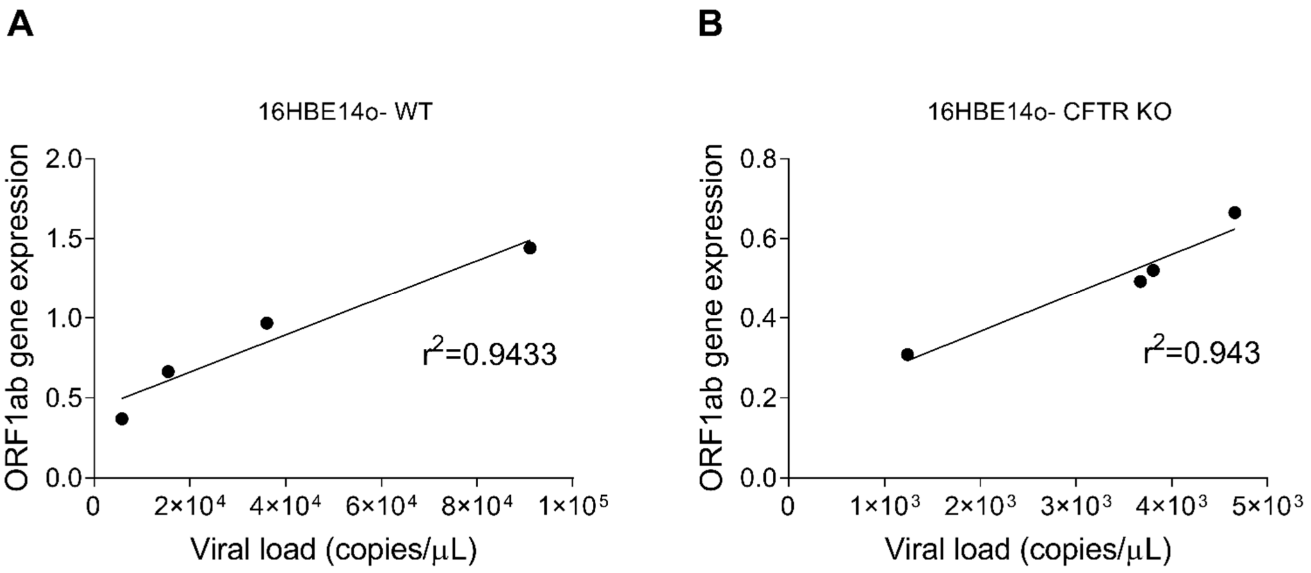

Supplementary Figure S1. SARS-CoV-2 ORF1ab gene expression and viral load correlation analysis. Regression curve of SARS-CoV-2 ORF1ab gene expression and viral load in (A) 16HBE14o- WT and (B) CFTR KO cells. The graphs represent the linear correlation of the different time points analyzed.

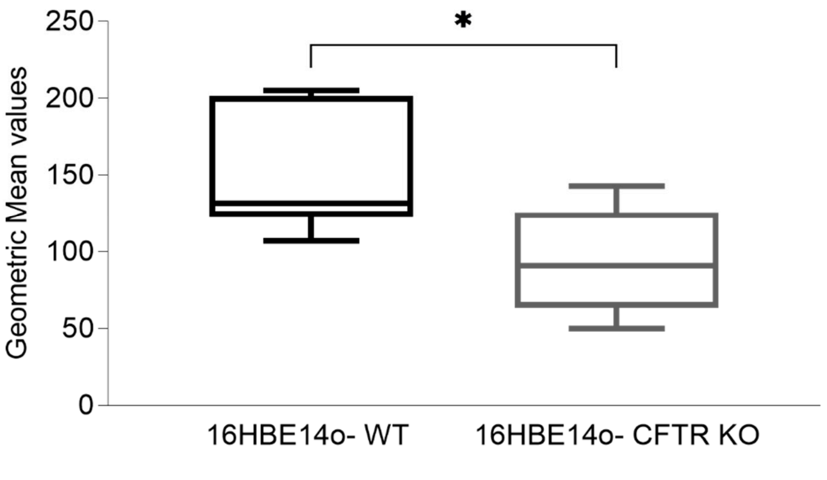

Supplementary Figure S2. Flow cytometry analysis of ACE-2 cell membrane expression in mock-infected CFTR WT and KO 16HBE14o- cells. Cells were marked with an ACE-2 antibody (bs-1004R, Bioss, Woburn, MA, USA, 1:150 dilution) and probed with an Alexa Fluor 488-conjugated secondary antibody (10 µg/mL). Analysis was performed using a MACS Quant10 (Miltenyi Biotec GmbH, Bergisch Gladbach, Germany), and data were analyzed using FlowJo™ v.10.7.1. software (BD Life Sciences, Franklin Lakes, NJ, USA). Data are reported as geometric mean values ± SD of independent experiments (n=3; * $p < 0.05$).

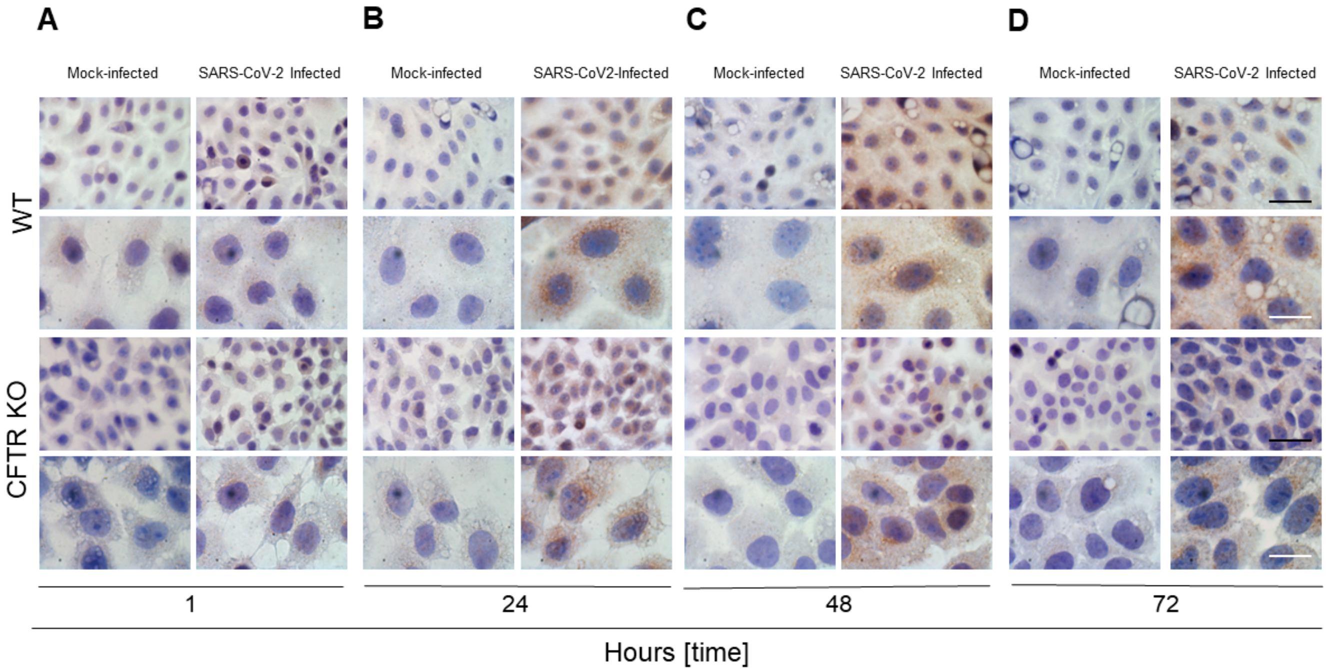

Supplementary Figure S3. Immunohistochemical analysis of SARS-CoV-2 N protein. Both mock- and SARS-CoV-2-infected WT and CFTR KO 16HBE14o- cells were stained with SARS Nucleocapsid Protein Antibody. (A) SARS-CoV-2 nucleocapsid protein immunoreactivity in mock- and SARS-CoV-2-infected WT and CFTR KO 16HBE14o- cells after 1 hour of culture. (B) SARS-CoV-2 nucleocapsid protein immunoreactivity in mock- and SARS-CoV-2-infected WT and CFTR KO 16HBE14o- cells after 24 hours of culture. (C) SARS-CoV-2 nucleocapsid protein immunoreactivity in mock- and SARS-CoV-2-infected WT and CFTR KO 16HBE14o- cells after 48 hours of culture. (D) SARS-CoV-2 nucleocapsid protein immunoreactivity in mock- and SARS-CoV-2-infected WT and CFTR KO 16HBE14o- cells after 72 hours of culture. White bars: 5 μm; black bars: 12 μm.

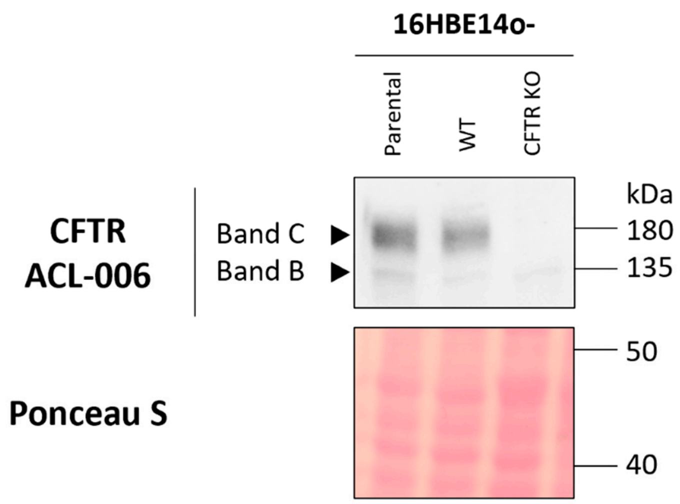

Supplementary Figure S4. Expression of CFTR protein. Representative Western Blot analysis of CFTR protein expression in 16HBE14o- cells expressing WT or KO CFTR. CFTR was detected using the anti-CFTR ACL-006 (Alomone Labs, Jerusalem, Israel, 1:1000 dilution). Ponceau S Red staining (~40-50kDa) reported relative protein band intensity. Chemiluminescence signals were acquired by ImageQuantTM LAS 4000 (GE Healthcare Europe GmbH, Milan, Italy).
